# Indole-3-thio­uronium iodide

**DOI:** 10.1107/S1600536807064719

**Published:** 2007-12-06

**Authors:** Martin Lutz, Anthony L. Spek, Erwin P. L. van der Geer, Gerard van Koten, Robertus J. M. Klein Gebbink

**Affiliations:** a Crystal and Structural Chemistry, Bijvoet Center for Biomolecular Research, Faculty of Science, Utrecht University, Padualaan 8, 3584 CH Utrecht, The Netherlands; b Chemical Biology & Organic Chemistry, Faculty of Science, Utrecht University, Padualaan 8, 3584 CH Utrecht, The Netherlands

## Abstract

In the title compound, C_9_H_10_N_3_S^+^·I^−^, the indole ring system and the thiouronium group are essentially perpendicular, with a dihedral angle of 89.87 (8)°. By inter­molecular hydrogen bonding, a three-dimensional network is formed, which is additionally supported by inter­molecular C—H⋯π inter­actions.

## Related literature

For the synthesis of the title compound, see: Harris (1969 ▶); van der Geer *et al.* (2007 ▶). For the crystal structures of similar compounds, see: Lutz *et al.* (2008 ▶); Ng (1995 ▶). For the characterization of C—H⋯π inter­actions, see: Malone *et al.* (1997 ▶). For thermal-motion analysis, see: Schomaker & Trueblood (1998 ▶). For the Cambridge Structural Database (update of August 2007), see: Allen (2002 ▶).
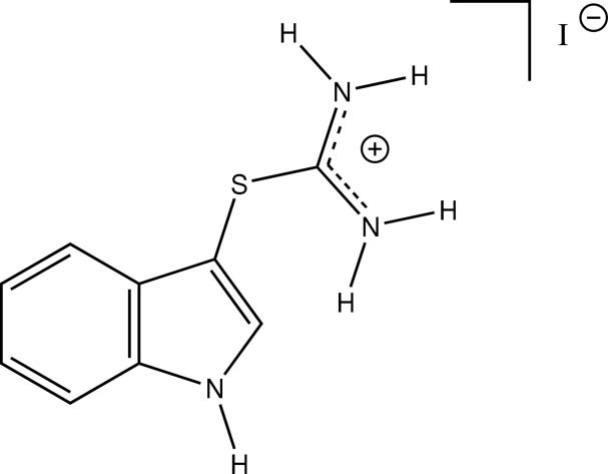


## Experimental

### 

#### Crystal data

C_9_H_10_N_3_S^+^·I^−^
*M* *_r_* = 319.16
Monoclinic, 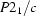

*a* = 10.5098 (2) Å
*b* = 10.6264 (3) Å
*c* = 10.6951 (4) Å
β = 102.648 (2)°
*V* = 1165.46 (6) Å^3^
*Z* = 4
Mo *K*α radiation
μ = 2.89 mm^−1^
*T* = 150 (2) K
0.30 × 0.30 × 0.30 mm

#### Data collection

Nonius KappaCCD diffractometer
Absorption correction: multi-scan (*SADABS*; Sheldrick, 2002 ▶) *T*_min_ = 0.24, *T*_max_ = 0.42
15531 measured reflections
2668 independent reflections
2470 reflections with *I* > 2σ(*I*)
*R*_int_ = 0.033

#### Refinement

*R*[*F* ^2^ > 2σ(*F* ^2^)] = 0.018
*wR*(*F* ^2^) = 0.045
*S* = 1.09
2668 reflections
167 parameters
All H-atom parameters refined
Δρ_max_ = 0.50 e Å^−3^
Δρ_min_ = −0.53 e Å^−3^

### 

Data collection: *COLLECT* (Nonius, 1999 ▶); cell refinement: *PEAKREF* (Schreurs, 2005 ▶); data reduction: *EVAL14* (Duisenberg *et al.*, 2003 ▶) and *SADABS* (Sheldrick, 2002 ▶); program(s) used to solve structure: *SHELXS97* (Sheldrick, 1997 ▶); program(s) used to refine structure: *SHELXL97* (Sheldrick, 1997 ▶); molecular graphics: *PLATON* (Spek, 2003 ▶); software used to prepare material for publication: *SHELXL97*.

## Supplementary Material

Crystal structure: contains datablocks I, global. DOI: 10.1107/S1600536807064719/bt2659sup1.cif

Structure factors: contains datablocks I. DOI: 10.1107/S1600536807064719/bt2659Isup2.hkl

Additional supplementary materials:  crystallographic information; 3D view; checkCIF report

## Figures and Tables

**Table 1:** Hydrogen-bond geometry (Å, °)

*D*—H⋯*A*	*D*—H	H⋯*A*	*D*⋯*A*	*D*—H⋯*A*
N1—H1*N*⋯I1^i^	0.76 (2)	2.91 (2)	3.6295 (17)	158 (2)
N2—H2*N*⋯I1	0.86 (3)	2.76 (3)	3.5736 (18)	158 (2)
N2—H3*N*⋯I1^ii^	0.80 (2)	2.97 (2)	3.6269 (17)	141 (2)
N3—H4*N*⋯I1^iii^	0.75 (3)	2.86 (3)	3.5990 (19)	165 (2)
N3—H5*N*⋯I1	0.88 (3)	2.95 (3)	3.7258 (19)	149 (2)
C1—H1⋯*Cg*1^iv^	0.91 (2)	2.91 (2)	3.794 (2)	162.8 (18)

Symmetry codes: (i) 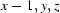; (ii) 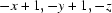; (iii) 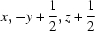; (iv) 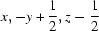. *Cg*1 is the centroid of the six-membered ring.

**Fig. 1.**
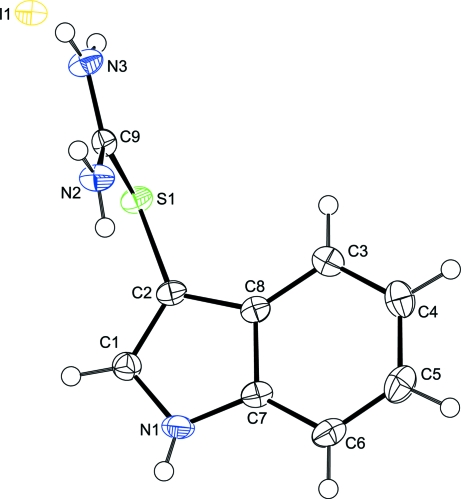
: The molecular structure of (I). Displacement ellipsoids are drawn at the 50% probability level and H atoms are shown as small spheres of arbitrary radii.

**Fig. 2.**
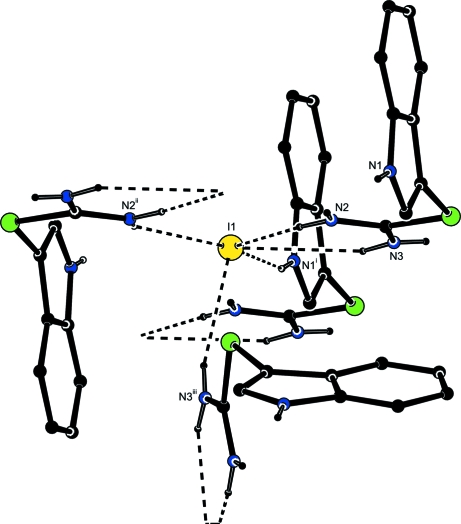
: Hydrogen bonded environment of the iodide in (I). C—H hydrogen atoms are omitted for clarity. Symmetry operations i: 1 + x, y, z; ii: 1 - x, 1 - y, -z; iii: x, 0.5 - y, z - 1/2.

**Fig. 3.**
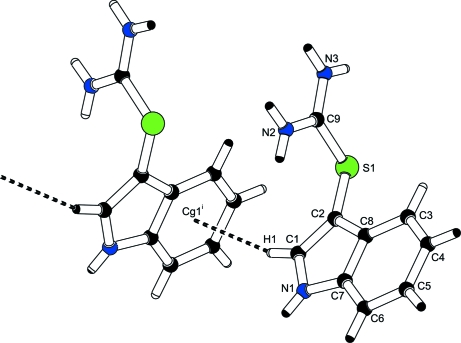
: C—H···π interaction in (I). View along the crystallographic b axis. Symmetry operation i: x, 0.5 - y, z - 1/2.

## supplementary crystallographic information

### Comment

Uronium and thiouronium ions are positively charged with the charge delocalized over the N—C—N group. In crystal engineering this group is therefore complementary to the negatively charged carboxylate group, not only in charge distribution but also in hydrogen-bonding ability.

The molecular geometry of the cation of title compound indole-3-thiouronium iodide (I) (Fig. 1) is very similar to the corresponding nitrate salt (Lutz *et al.*, 2007). The C—N bond lengths of 1.306 (2) and 1.317 (2) Å show significant double bond character while the C—S bond of 1.7533 (19) Å is in the expected range for a single bond. Similar distances and angles have also been found in the benzylthiouronium cation (Ng, 1995).

As in the nitrate salt, the cation consists of two planar subunits, *i.e.* the indole and the thiouronium moieties, which are perpendicular to each other with an angle of 89.87 (8)° between the corresponding least squares planes. The weighted *R* value of a thermal motion analysis using the program THMA11 (Schomaker & Trueblood, 1998) results in a low weighted *R* value of 0.106, which is slightly higher than in the nitrate salt (0.084).

The iodide anion is surrounded by five N—H groups which act as hydrogen bond donors (Fig. 2). This results in a three dimensional hydrogen bonded network. The H···I distances of 2.76 (3) to 2.97 (2) Å are in the same range as found for other N—H···I hydrogen bonds in the Cambridge Structural Database (update August 2007; Allen, 2002), where we calculate an average H···I distance of 2.80 Å. In general, N—H···I hydrogen bonds are relatively weak; the average hydrogen bonded intermolecular N···I distance is 3.65 Å in the Cambridge Structural Database, which is not shorter than the sum of van der Waals radii of 1.55 (nitrogen) plus 1.98 Å (iodine).

In addition to the N—H···I hydrogen bonds there are weak intermolecular C—H···π interactions between H1 and the six-membered ring of the indole moiety (Fig. 3). The distance of H1 to the least squares plane of the six-membered ring is 2.83 (2) Å and the distance to the center of gravity of this ring is 2.91 (2) Å (Table 2). According to the classification of Malone *et al.* (1997) this is a "Type I" C—H···π interaction.

### Experimental

Indole-3-thiouronium iodide was prepared as described in literature (Harris, 1969; van der Geer *et al.*, 2007) and crystallized by diethyl ether vapor diffusion into an acetone solution.

### Refinement

All H atoms were freely refined.

### Crystal data

**Table:**

C_9_H_10_N_3_S^+^·I^–^	*F*_000_ = 616
*M**_r_* = 319.16	*D*_x_ = 1.819 Mg m^−^^3^
Monoclinic, *P*2_1_/*c*	Mo *K*α radiation λ = 0.71073 Å
Hall symbol: -P 2ybc	Cell parameters from 11915 reflections
*a* = 10.5098 (2) Å	θ = 2.0–27.5º
*b* = 10.6264 (3) Å	µ = 2.89 mm^−^^1^
*c* = 10.6951 (4) Å	*T* = 150 (2) K
β = 102.648 (2)º	Block, colourless
*V* = 1165.46 (6) Å^3^	0.30 × 0.30 × 0.30 mm
*Z* = 4	

### Data collection

**Table:**

Nonius KappaCCD diffractometer	2668 independent reflections
Radiation source: rotating anode	2470 reflections with *I* > 2σ(*I*)
Monochromator: graphite	*R*_int_ = 0.033
*T* = 150(2) K	θ_max_ = 27.5º
φ and ω scans	θ_min_ = 2.0º
Absorption correction: multi-scan(SADABS; Sheldrick, 2002)	*h* = −13→13
*T*_min_ = 0.24, *T*_max_ = 0.42	*k* = −13→13
15531 measured reflections	*l* = −13→13

### Refinement

**Table:**

Refinement on *F*^2^	Secondary atom site location: difference Fourier map
Least-squares matrix: full	Hydrogen site location: difference Fourier map
*R*[*F*^2^ > 2σ(*F*^2^)] = 0.018	All H-atom parameters refined
*wR*(*F*^2^) = 0.045	*w* = 1/[σ^2^(*F*_o_^2^) + (0.0216*P*)^2^ + 0.5241*P*] where *P* = (*F*_o_^2^ + 2*F*_c_^2^)/3
*S* = 1.09	(Δ/σ)_max_ = 0.003
2668 reflections	Δρ_max_ = 0.50 e Å^−^^3^
167 parameters	Δρ_min_ = −0.53 e Å^−^^3^
Primary atom site location: structure-invariant direct methods	Extinction correction: none

### Special details

**Table:**

Geometry. All e.s.d.'s (except the e.s.d. in the dihedral angle between two l.s. planes) are estimated using the full covariance matrix. The cell e.s.d.'s are taken into account individually in the estimation of e.s.d.'s in distances, angles and torsion angles; correlations between e.s.d.'s in cell parameters are only used when they are defined by crystal symmetry. An approximate (isotropic) treatment of cell e.s.d.'s is used for estimating e.s.d.'s involving l.s. planes.
Refinement. Refinement of *F*^2^ against ALL reflections. The weighted *R*-factor *wR* and goodness of fit *S* are based on *F*^2^, conventional *R*-factors *R* are based on *F*, with *F* set to zero for negative *F*^2^. The threshold expression of *F*^2^ > σ(*F*^2^) is used only for calculating *R*-factors(gt) *etc*. and is not relevant to the choice of reflections for refinement. *R*-factors based on *F*^2^ are statistically about twice as large as those based on *F*, and *R*- factors based on ALL data will be even larger.

### Fractional atomic coordinates and isotropic or equivalent isotropic displacement parameters (Å^2^)

**Table:**

	*x*	*y*	*z*	*U*_iso_*/*U*_eq_	
S1	0.43028 (5)	0.17217 (4)	0.29661 (4)	0.02089 (10)	
N1	0.08974 (16)	0.33441 (15)	0.18625 (17)	0.0243 (3)	
H1N	0.024 (2)	0.343 (2)	0.140 (2)	0.017 (5)*	
N2	0.49115 (18)	0.34694 (16)	0.13906 (16)	0.0237 (3)	
H2N	0.541 (3)	0.384 (3)	0.097 (2)	0.039 (7)*	
H3N	0.415 (2)	0.365 (2)	0.123 (2)	0.025 (6)*	
N3	0.65635 (17)	0.21727 (17)	0.23827 (18)	0.0247 (3)	
H4N	0.680 (2)	0.170 (2)	0.291 (2)	0.025 (6)*	
H5N	0.711 (2)	0.251 (2)	0.196 (3)	0.040 (7)*	
C1	0.18173 (18)	0.24948 (18)	0.17078 (18)	0.0220 (4)	
H1	0.168 (2)	0.198 (2)	0.101 (2)	0.032 (6)*	
C2	0.29152 (17)	0.26651 (17)	0.26602 (17)	0.0193 (3)	
C3	0.3378 (2)	0.42952 (18)	0.45307 (19)	0.0238 (4)	
H3	0.422 (2)	0.402 (2)	0.491 (2)	0.022 (5)*	
C4	0.2801 (2)	0.52789 (19)	0.5048 (2)	0.0287 (4)	
H4	0.325 (3)	0.574 (3)	0.577 (3)	0.047 (8)*	
C5	0.1524 (2)	0.5675 (2)	0.4494 (2)	0.0312 (5)	
H5	0.117 (2)	0.635 (2)	0.487 (2)	0.027 (6)*	
C6	0.0801 (2)	0.5097 (2)	0.3418 (2)	0.0277 (4)	
H6	−0.004 (3)	0.536 (2)	0.304 (3)	0.034 (7)*	
C7	0.13841 (18)	0.40987 (18)	0.29061 (18)	0.0218 (4)	
C8	0.26581 (17)	0.36941 (16)	0.34426 (17)	0.0189 (3)	
C9	0.53403 (17)	0.25457 (16)	0.21755 (16)	0.0183 (3)	
I1	0.770943 (11)	0.454617 (11)	0.031246 (11)	0.02163 (5)	

### Atomic displacement parameters (Å^2^)

**Table:**

	*U*^11^	*U*^22^	*U*^33^	*U*^12^	*U*^13^	*U*^23^
S1	0.0194 (2)	0.0196 (2)	0.0242 (2)	0.00360 (16)	0.00591 (17)	0.00584 (17)
N1	0.0146 (8)	0.0273 (8)	0.0288 (9)	0.0010 (6)	−0.0001 (7)	−0.0015 (7)
N2	0.0188 (8)	0.0262 (8)	0.0259 (8)	0.0001 (7)	0.0045 (7)	0.0077 (6)
N3	0.0202 (8)	0.0272 (9)	0.0278 (9)	0.0059 (7)	0.0076 (7)	0.0069 (7)
C1	0.0210 (9)	0.0227 (9)	0.0222 (9)	−0.0008 (7)	0.0047 (7)	−0.0001 (7)
C2	0.0160 (8)	0.0200 (8)	0.0227 (9)	0.0001 (7)	0.0058 (7)	0.0032 (7)
C3	0.0238 (10)	0.0234 (9)	0.0232 (9)	−0.0020 (7)	0.0032 (8)	0.0034 (7)
C4	0.0367 (13)	0.0238 (10)	0.0251 (10)	−0.0043 (8)	0.0059 (9)	−0.0023 (8)
C5	0.0365 (13)	0.0231 (10)	0.0378 (12)	0.0020 (8)	0.0165 (10)	−0.0032 (8)
C6	0.0219 (10)	0.0273 (10)	0.0354 (11)	0.0049 (8)	0.0094 (9)	0.0003 (8)
C7	0.0180 (9)	0.0211 (8)	0.0266 (9)	0.0004 (7)	0.0058 (7)	0.0023 (7)
C8	0.0185 (9)	0.0184 (8)	0.0210 (8)	−0.0006 (7)	0.0067 (7)	0.0042 (7)
C9	0.0198 (9)	0.0175 (8)	0.0173 (8)	−0.0007 (7)	0.0031 (6)	−0.0014 (6)
I1	0.01614 (8)	0.02314 (8)	0.02444 (8)	0.00029 (4)	0.00192 (5)	0.00236 (4)

### Geometric parameters (Å, °)

**Table:**

S1—C2	1.7406 (18)	C1—H1	0.91 (2)
S1—C9	1.7533 (19)	C2—C8	1.438 (2)
N1—C1	1.359 (2)	C3—C4	1.383 (3)
N1—C7	1.379 (3)	C3—C8	1.396 (3)
N1—H1N	0.76 (2)	C3—H3	0.94 (2)
N2—C9	1.306 (2)	C4—C5	1.407 (4)
N2—H2N	0.86 (3)	C4—H4	0.95 (3)
N2—H3N	0.80 (2)	C5—C6	1.377 (3)
N3—C9	1.317 (2)	C5—H5	0.94 (2)
N3—H4N	0.75 (3)	C6—C7	1.396 (3)
N3—H5N	0.88 (3)	C6—H6	0.93 (3)
C1—C2	1.374 (3)	C7—C8	1.404 (3)
			
C2—S1—C9	101.87 (9)	C3—C4—C5	121.3 (2)
C1—N1—C7	109.70 (16)	C3—C4—H4	122.2 (18)
C1—N1—H1N	124.2 (16)	C5—C4—H4	116.5 (18)
C7—N1—H1N	125.7 (16)	C6—C5—C4	121.3 (2)
C9—N2—H2N	121.1 (18)	C6—C5—H5	119.9 (14)
C9—N2—H3N	120.1 (17)	C4—C5—H5	118.8 (14)
H2N—N2—H3N	118 (2)	C5—C6—C7	117.18 (19)
C9—N3—H4N	118.3 (18)	C5—C6—H6	121.6 (15)
C9—N3—H5N	120.9 (17)	C7—C6—H6	121.2 (15)
H4N—N3—H5N	121 (2)	N1—C7—C6	129.98 (18)
N1—C1—C2	109.07 (17)	N1—C7—C8	107.73 (16)
N1—C1—H1	120.7 (15)	C6—C7—C8	122.29 (18)
C2—C1—H1	130.0 (15)	C3—C8—C7	119.67 (17)
C1—C2—C8	107.29 (17)	C3—C8—C2	134.13 (18)
C1—C2—S1	126.63 (15)	C7—C8—C2	106.20 (16)
C8—C2—S1	125.81 (14)	N2—C9—N3	121.41 (18)
C4—C3—C8	118.29 (19)	N2—C9—S1	121.45 (15)
C4—C3—H3	121.6 (14)	N3—C9—S1	117.12 (14)
C8—C3—H3	120.1 (14)		
			
C7—N1—C1—C2	1.0 (2)	C4—C3—C8—C7	0.1 (3)
N1—C1—C2—C8	−0.6 (2)	C4—C3—C8—C2	−179.7 (2)
N1—C1—C2—S1	173.64 (14)	N1—C7—C8—C3	−179.32 (17)
C9—S1—C2—C1	96.99 (19)	C6—C7—C8—C3	0.4 (3)
C9—S1—C2—C8	−89.75 (17)	N1—C7—C8—C2	0.5 (2)
C8—C3—C4—C5	−0.3 (3)	C6—C7—C8—C2	−179.74 (18)
C3—C4—C5—C6	0.1 (3)	C1—C2—C8—C3	179.9 (2)
C4—C5—C6—C7	0.4 (3)	S1—C2—C8—C3	5.5 (3)
C1—N1—C7—C6	179.4 (2)	C1—C2—C8—C7	0.1 (2)
C1—N1—C7—C8	−0.9 (2)	S1—C2—C8—C7	−174.27 (14)
C5—C6—C7—N1	179.0 (2)	C2—S1—C9—N2	−11.53 (17)
C5—C6—C7—C8	−0.7 (3)	C2—S1—C9—N3	170.10 (14)

### Hydrogen-bond geometry (Å, °)

**Table:**

*D*—H···*A*	*D*—H	H···*A*	*D*···*A*	*D*—H···*A*
N1—H1N···I1^i^	0.76 (2)	2.91 (2)	3.6295 (17)	158 (2)
N2—H2N···I1	0.86 (3)	2.76 (3)	3.5736 (18)	158 (2)
N2—H3N···I1^ii^	0.80 (2)	2.97 (2)	3.6269 (17)	141 (2)
N3—H4N···I1^iii^	0.75 (3)	2.86 (3)	3.5990 (19)	165 (2)
N3—H5N···I1	0.88 (3)	2.95 (3)	3.7258 (19)	149 (2)
C1—H1···Cg1^iv^	0.91 (2)	2.91 (2)	3.794 (2)	162.8 (18)

Symmetry codes: (i) *x*−1, *y*, *z*; (ii) −*x*+1, −*y*+1, −*z*; (iii) *x*, −*y*+1/2, *z*+1/2; (iv) *x*, −*y*+1/2, *z*−1/2.
